# Supplementary material for: COVID-19 diagnosis by routine blood tests using machine learning
Source: Sci Rep. 2021 May 24;11:10738. doi: 10.1038/s41598-021-90265-9 (PMC8144373; doi:10.1038/s41598-021-90265-9)
Supplement: Supplementary file 1 — Supplementary Information. [file 41598_2021_90265_MOESM1_ESM.pdf]

COVID-19 diagnosis by routine blood tests using machine learning

Matjaž Kukar,[1,2*] Gregor Gunčar,[1,3*] Tomaž Vovko,[4] Simon Podnar,[5] Peter Černelč,[7] Miran Brvar,[6] Mateja Zalaznik,[4] Mateja Notar,[1] Sašo Moškon,[1] Marko Notar[#1]

[1]Smart Blood Analytics Swiss SA, CH-8008 Zürich, Switzerland
[2]Faculty of Computer and Information Science, University of Ljubljana, Slovenia
[3]Faculty of Chemistry and Chemical Technology, University of Ljubljana, Slovenia
[4]Department of Infectious Diseases, University Medical Centre Ljubljana, Slovenia
[5]Division of Neurology, University Medical Centre Ljubljana, Slovenia
[6]Centre for Clinical Toxicology and Pharmacology, University Medical Centre Ljubljana, Slovenia
[7]Division of Internal Medicine, University Medical Centre Ljubljana, Slovenia
[*]Joint first authors, contributed equally.

[#]Corresponding author marko@sba-swiss.com

Supplementary Table S1 Blood Parameters, sex, and age values used in the COVID-19 ML model. For each parameter, its mean, standard deviation, mean and interquartile range is given separately for: BAC- bacterial, VIR- viral (excluding COVID-19) or COVID- COVID-19 infection.

parameter	unit	disease	median	mean	st. dev.	inter-quartile range
Leukocyte count	1E9/L	COVID	5.150	5.829	2.827	4-6.9
		BAC	9.100	10.843	7.085	6.6-13.3
		VIR	6.100	6.367	4.015	4.5-7.7
Erythrocyte count	1E12/L	COVID	4.830	4.693	0.636	4.38-5.07
		BAC	3.860	3.853	0.746	3.35-4.36
		VIR	4.540	4.453	0.656	4.07-4.91
Hemoglobin	g/L	COVID	144.500	141.769	19.722	131-153
		BAC	116.000	115.406	21.835	100-130
		VIR	137.000	134.854	18.581	123-148
Hematocrit	1	COVID	0.421	0.415	0.055	0.385-0.447
		BAC	0.345	0.345	0.064	0.304-0.388
		VIR	0.413	0.407	0.053	0.375-0.444
Mean Corpuscular Volume	fL	COVID	88.900	88.543	4.537	86-90.8
		BAC	89.700	90.045	7.096	86-93.9
		VIR	91.600	91.760	6.028	88.4-95
Mean Corpuscular Hemoglobin	pg/cell	COVID	30.300	30.238	1.677	29.4-31.3
		BAC	30.100	30.065	2.622	28.6-31.5
		VIR	30.500	30.410	2.220	29.3-31.7
Mean Corpuscular Hemoglobin Concentration	g/L	COVID	342.500	342.344	9.763	339-347
		BAC	334.000	333.773	14.496	325-343
		VIR	332.000	331.376	9.410	325-337
Erythrocyte Distribution Width	1	COVID	0.134	0.139	0.028	0.129-0.141
		BAC	0.148	0.152	0.024	0.138-0.163
		VIR	0.134	0.139	0.018	0.128-0.145
Thrombocytes count	1E9/L	COVID	175.000	191.163	78.434	146-222
		BAC	218.000	234.925	115.945	157-291
		VIR	174.000	176.393	74.149	126-220
Mean Platelet Volume	fL	COVID	9.100	9.236	0.943	8.6-9.8
		BAC	8.700	8.855	1.286	8-9.6
		VIR	8.700	8.782	1.038	8.1-9.4
Lymphocyte count	1E9/L	COVID	1.200	1.327	0.718	0.8-1.6
		BAC	1.000	1.328	2.761	0.6-1.51
		VIR	1.700	1.876	2.528	1.2-2.3
Monocyte count	1E9/L	COVID	0.405	0.520	0.325	0.34-0.617
		BAC	0.500	0.571	0.449	0.3-0.7
		VIR	0.500	0.538	0.318	0.4-0.7
Neutrophils count	1E9/L	COVID	3.155	3.811	2.483	2.2-4.78
		BAC	6.600	7.820	5.156	4.4-9.8
		VIR	3.400	3.721	2.184	2.4-4.6
Eosinophils count	1E9/L	COVID	0.010	0.041	0.062	0-0.07
		BAC	0.100	0.159	0.222	0.02-0.2
		VIR	0.100	0.142	0.140	0.1-0.2
Basophils count	1E9/L	COVID	0.010	0.016	0.024	0-0.02
		BAC	0.000	0.035	0.076	0-0.1
		VIR	0.000	0.031	0.051	0-0.1

parameter	unit	disease	median	mean	st. dev.	inter-quartile range
**Lymphocyte %**	**1**	**COVID**	0.235	0.254	0.121	0.165-0.336
		**BAC**	0.118	0.141	0.108	0.0607-0.195
		**VIR**	0.299	0.303	0.107	0.236-0.367
**Monocyte %**	**1**	**COVID**	0.096	0.108	0.059	0.073-0.136
		**BAC**	0.066	0.066	0.034	0.04-0.088
		**VIR**	0.086	0.090	0.032	0.07-0.106
**Neutrophils %**	**1**	**COVID**	0.640	0.622	0.149	0.516-0.734
		**BAC**	0.756	0.728	0.157	0.66-0.83
		**VIR**	0.578	0.574	0.120	0.501-0.651
**Eosinophils %**	**1**	**COVID**	0.005	0.008	0.010	0.00125-0.012
		**BAC**	0.012	0.019	0.025	0.006-0.025
		**VIR**	0.017	0.023	0.020	0.01-0.029
**Basophils %**	**1**	**COVID**	0.004	0.004	0.003	0.002-0.006
		**BAC**	0.004	0.005	0.005	0.002-0.007
		**VIR**	0.006	0.006	0.004	0.004-0.009
**Prothrombin activity %**	**1**	**COVID**	1.050	1.022	0.187	0.99-1.14
		**BAC**	0.830	0.755	0.297	0.55-0.99
		**VIR**	0.910	0.884	0.187	0.81-1
**Prothrombin international normalized ratio**	**1**	**COVID**	0.960	1.010	0.232	0.92-1.01
		**BAC**	1.120	1.535	1.285	1-1.41
		**VIR**	1.060	1.128	0.385	1-1.13
**Glucose**	**mmol/L**	**COVID**	5.400	6.214	2.613	4.9-6.32
		**BAC**	6.250	7.561	4.671	5.1-8.3
		**VIR**	4.900	5.322	1.789	4.5-5.6
**Urea**	**mmol/L**	**COVID**	5.300	6.644	4.500	4.2-7.6
		**BAC**	14.200	17.909	13.447	7.8-24.1
		**VIR**	5.000	5.634	3.117	4.1-6.2
**Creatinine**	**µmol/L**	**COVID**	85.000	97.696	61.215	71-102
		**BAC**	158.000	248.429	235.189	87-330
		**VIR**	69.000	78.905	65.978	60-80
**Bilirubin total**	**µmol/L**	**COVID**	9.000	10.794	8.161	7-12
		**BAC**	10.000	17.223	32.298	7-16
		**VIR**	11.000	18.169	36.088	8-16
**Alkaline Phosphatase**	**µkat/L**	**COVID**	1.080	1.152	0.366	0.9-1.29
		**BAC**	1.420	1.877	1.643	1.04-2.11
		**VIR**	1.230	1.470	1.081	0.99-1.58
**Bilirubin direct**	**µmol/L**	**COVID**	3.000	4.039	3.263	2-5
		**BAC**	4.000	9.493	24.331	2-8
		**VIR**	4.000	8.522	26.014	3-6
**Potassium in serum**	**mmol/L**	**COVID**	4.200	4.210	0.464	3.9-4.5
		**BAC**	4.470	4.568	0.876	4-5
		**VIR**	4.200	4.252	0.384	4-4.5
**Sodium in serum**	**mmol/L**	**COVID**	139.000	138.525	3.001	137-140
		**BAC**	139.000	138.731	5.923	136-142
		**VIR**	141.000	140.577	2.862	139-142
**Alanine Aminotransferase**	**µkat/L**	**COVID**	0.540	0.632	0.408	0.4-0.7
		**BAC**	0.410	0.994	3.956	0.28-0.72
		**VIR**	0.600	1.430	4.499	0.39-1.08
**Aspartate Aminotransferase**	**µkat/L**	**COVID**	0.470	0.640	0.587	0.37-0.67
		**BAC**	0.460	1.272	4.798	0.32-0.82
		**VIR**	0.520	1.002	2.693	0.39-0.83
**Gamma-Glutamyltransferase**	**µkat/L**	**COVID**	0.380	0.668	0.914	0.24-0.692
		**BAC**	0.700	1.515	2.331	0.37-1.6
		**VIR**	0.530	1.130	1.878	0.32-1.11
**Albumin**	**g/L**	**COVID**	41.000	39.726	5.868	36.2-44
		**BAC**	34.000	33.516	7.244	28-39
		**VIR**	43.000	42.417	5.145	41-46
**C-Reactive Protein**	**mg/L**	**COVID**	12.000	40.700	61.102	4.5-49.2
		**BAC**	52.000	90.683	97.894	9-147
		**VIR**	4.500	9.299	19.417	4.5-4.5
**age**	**years**	**COVID**	55.500	53.331	18.804	38-67.2
		**BAC**	70.000	66.547	16.894	56-80
		**VIR**	47.000	48.553	13.889	38-59
**sex**		**COVID**	female	41.9%	male	58.1%
		**BAC**	female	44.5%	male	55.5%
		**VIR**	female	37.2%	male	62.8%
